# Pre-Existing Vector Immunity Does Not Prevent Replication Deficient Adenovirus from Inducing Efficient CD8 T-Cell Memory and Recall Responses

**DOI:** 10.1371/journal.pone.0034884

**Published:** 2012-04-13

**Authors:** Maria Abildgaard Steffensen, Benjamin Anderschou Holbech Jensen, Peter Johannes Holst, Maria Rosaria Bassi, Jan Pravsgaard Christensen, Allan Randrup Thomsen

**Affiliations:** Institute of International Health, Immunology, and Microbiology, University of Copenhagen, Copenhagen, Denmark; Massachusetts General Hospital, United States of America

## Abstract

Adenoviral vectors have shown a great potential for vaccine development due to their inherent ability to induce potent and protective CD8 T-cell responses. However, a critical issue regarding the use of these vectors is the existence of inhibitory immunity against the most commonly used Ad5 vector in a large part of the human population. We have recently developed an improved adenoviral vaccine vector system in which the vector expresses the transgene tethered to the MHC class II associated invariant chain (Ii). To further evaluate the potential of this system, the concept of pre-existing inhibitory immunity to adenoviral vectors was revisited to investigate whether the inhibition previously seen with the Ad5 vector also applied to the optimized vector system. We found this to be the case, and antibodies dominated as the mechanism underlying inhibitory vector immunity. However, presence of CD8 T cells directed against epitopes in the adenoviral vector seemed to correlate with repression of the induced response in re-vaccinated B-cell deficient mice. More importantly, despite a repressed primary effector CD8 T-cell response in Ad5-immune animals subjected to vaccination, memory T cells were generated that provided the foundation for an efficient recall response and protection upon subsequent viral challenge. Furthermore, the transgene specific response could be efficiently boosted by homologous re-immunization. Taken together, these studies indicate that adenoviral vectors can be used to induce efficient CD8 T-cell memory even in individuals with pre-existing vector immunity.

## Introduction

Development of effective vaccines against diseases such as HIV, hepatitis C and cancer will require vaccine formulations capable of inducing effective and long lasting cellular immunity. Most current effective vaccines are protective primarily due to the induction of antibodies against the targeted pathogen. However, several diseases cannot be effectively prevented or treated by an antibody response alone, and at present time, vaccines which are effective against these types of diseases do not exist. There are only few vaccine vectors capable of inducing efficient and long lasting cellular immunity, and one of these is the replication-incompetent adenovirus serotype 5 vector (Ad5). However, a major issue concerning the use of this vector is that there have been several reports from experimental studies indicating that prior contact with this virus may inhibit the generation of an immune response induced through vaccination [1]–[5]. Since a large fraction of the human population has encountered Ad5 naturally, they will also have pre-existing Ad5 immunity [6], [7]. Moreover, even if the use of vaccine vectors based on other adenoviral serotypes should become common practice in order to overcome this problem, the issue of pre-existing immunity is also relevant with regard to these serotypes. It is therefore extremely import to clearly define the extent to which pre-existing immunity may represent a barrier to adenoviral vaccination, the underlying mechanisms, and how inhibitory immunity may be circumvented.

Vaccination with a high dose of empty Ad5 vector has been shown to induce high titers of antibodies directed against the Ad5 capsid proteins [1]–[4], primarily the hexon protein [8], leading to a substantially inhibited primary immune response upon vaccination with antigens encoded by the Ad5 vector. However, it is not yet quite clear whether this inhibitory effect is also marked with regard to the generation of memory T cells and recall responses in Ad5 vaccinated individuals. Furthermore, various observations indicate that neutralizing antibodies are not the sole contributors to the observed inhibition [2], [3], [9], making the issue of inhibitory immunity more complex. Notably, previous studies have used the intramuscular route of vaccination, which might impact the results obtained from these studies, since muscle tissue has a high blood supply. On the one hand this could mean a greater risk of virus dissemination, but also that antibodies and immune cells would have more easy access to the site of vaccination. In this context, it is relevant to note that recent results from our group suggest that keeping the vector inoculums very localized as in the case of subcutaneous vaccination may in fact be a much better way to administer Ad vaccination [10]. It is therefore pertinent to establish whether the inhibitory effect of pre-existing immunity is just as pronounced when adenoviral vectors are given subcutaneously.

In the current study, we have used an optimized adenoviral vaccine vector system in which the vector expresses the glycoprotein (GP) of lymphocytic choriomeningitis virus (LCMV) tethered to the MHC class II associated invariant chain (Ii). This substantially enhances the CD8 T-cell response induced by the vector [11] and could therefore, in theory, also improve the vector's ability to overcome inhibitory immunity caused by prior encounter with the vector. It has recently been shown, that in the presence of pre-existing immunity, the branch of the cellular immune response that is most affected, is the CD8 T-cell response [12]. It is therefore of great importance to establish a vector system that is capable of overcoming this phenomenon, in order to create successful vaccine formulations capable of combating diseases such as HIV, hepatitis C and cancer, in which a potent and sustained CD8 T-cell response is crucial for elimination of virus infected or malignant cells.

For this reason, the concept of pre-existing inhibitory immunity to adenoviral vectors was revisited. While confirming earlier observations indicating that pre-existing antibodies represent the major suppressive factor [3], we also obtained results suggesting that adenovirus-specific T cells could play a significant role. More importantly, despite a repressed primary effector CD8 T-cell response in Ad-immune animals subjected to vaccination, memory T cells are still generated, which can efficiently provide the foundation for an improved immune response upon subsequent viral challenge. Additionally, in contrast to the existing literature [13], we observed efficient boosting of the transgene specific CD8 T-cell response following homologous re-immunization. Thus, there is every reason to assume that genetically modified adenoviruses may serve as efficient vaccine vectors inducing CD8 T-cell memory and protective immunity even in the presence of pre-existing antibodies to certain serotypes in a large percentage of the human population.

## Materials and Methods

### Ethics statement

Experiments were conducted in accordance with national Danish guidelines (Amendment # 1306 of November 23, 2007) regarding animal experiments as approved by the Danish Animal Experiments Inspectorate, Ministry of Justice, permission numbers 2004/561–867 and 2009/561–1679.

### Mice

Female C57BL/6 mice were obtained from Taconic Farms. B6.129S2-Igh-6tm1Cgn/J (B-cell deficient) mice were obtained from The Jackson Laboratory. All mice used in this study were about 8 weeks old when included in an experiment and housed in a specific pathogen-free facility.

### Adenoviral vectors and vaccination

Replication deficient E1-deleted Ad5 vectors with a non-functional E3 gene expressing either a truncated version of ovalbumin (Ova) or the GP of LCMV linked to Ii (designated Ad5-IiOva and Ad5-IiGP, respectively) were produced as described previously [14]. For production of the Ad35-IiGP vector, the IiGP insert was excised from the pacCMV plasmid via restriction enzymes and cloned into the pHMCMV5 plasmid (kindly provided by Hiroyuki Mizuguchi, Ph.D, Osaka University, Osaka, Japan), the shuttle vector for the Ad35 vector cloning system, that was originally developed by Mizuguchi and Kay [15], [16]. From the shuttle vector, the insert was cloned directly into the E1 deleted Ad35 backbone (kindly provided by Hiroyuki Mizuguchi, Ph.D) via homing endonucleases *PI-Sce I* and *I-Ceu I*. The Ad35-IiGP DNA was transfected into Hek-E1b cells (kindly provided by Hiroyuki Mizuguchi, Ph.D) to support production of virus particles. Adenoviral particles were purified using standard methods and aliquoted and frozen at −80°C in 10% glycerol. The IiGP insert sequence was verified by sequencing and restriction enzyme digestion. Infectivity of the adenovirus stocks was determined with the Adeno-X™ Rapid Titer Kit (Clontech Laboratories Inc., California, USA). The ratio of physical particles to infectious units for the adenoviral vaccines used in this study varied between 50 and 100.

In the majority of experiments mice to be vaccinated were anesthetized and injected with 2×10^7^ infectious units of Ad5 vector in 30 µl PBS s.c. in the hind foot pad; mice vaccinated using Ad35 vector received 5×10^8^ infectious unit of virus in the same manner. Mice to be vaccinated a second time received the injection in the contra lateral foot pad.

### Viruses

In addition to adenovirus, vaccinia virus and LCMV were utilized in this study. Vaccinia virus expressing the GP of LCMV (VV-GP) was kindly provided by A. Oxenius (ETH, Zürich, Switzerland). The virus was grown and titered using CV-1 cells. For infection of mice, a dose of 10^6^ pfu was given i.p. LCMV clone 13 was originally provided by M.B.A. Oldstone (The Scripps Research Institute, La Jolla, CA) and further propagated in-house using BHK cells. For infection of mice, a dose of 10^6^ pfu was given i.v.

### LCMV organ titers

Organs were homogenized as 10% organ suspensions, and viral titers were determined using an immune focus assay as previously described [17].

### Dendritic cell preparation

Femurs from mice were removed aseptically and purified from the surrounding tissue. The bone marrow was flushed out using a syringe filled with HBSS and single-cell suspensions were obtained by pressing the spleen through a fine steel mesh (70 µm), followed by centrifugation and resuspension in RPMI 1640 cell culture medium containing 10% FCS supplemented with NaHCO_3_, 2-ME, L-glutamine, and penicillin-streptomycin. 6×10^6^ cells were seeded in non-coated 175 cm^2^ cell culture flasks containing 30 ml cell medium supplemented with 20 ng/ml GM-CSF. Cell culture flasks were incubated at 37°C and 5% CO_2_, and were supplemented with fresh medium containing GM-CSF on days 3, 6 and 8 after cell purification [18]. On day 10, dendritic cells were washed and resuspended in medium containing 100 MOI (multiplicity of infection) of Ad5-IiGP. Cells and virus particles were incubated for 6 hours at 37°C and 5% CO_2_, followed by 3 washes and resuspension in PBS. Cell number was adjusted to 50×10^6^/ml and 100 µl were injected s.c. in the right flank of recipient mice.

### Flow cytometry

After vaccination of mice with Ad5-IiOva/GP or DCs, the frequencies of epitope specific CD8+ T cells were determined by intracellular cytokine staining after 5 h of incubation with relevant peptides (0.1 µg/ml GP33, GP276 or FALSNAEDL (CD8 T cell epitope in DBP of Ad5)) at 37°C and 5% CO_2_. After incubation, the cells were stained with Abs for cell surface markers (PerCP-Cy5.5-CD8 and FITC-CD44) and Ab for intracellular cytokine (APC-IFN-γ). Samples were run on FACSCalibur or LSRII flow cytometers (BD biosciences) and analyzed using Cellquest Pro (BD biosciences) or FlowJo software (Tree Star). Phenotype characterization of IFN-γ^+^ CD8 T cells was performed using the following markers: FITC-CD27, PE-Cy7-CD127 and PE-KLRG-1.

### Statistical evaluation

Data are presented as individual points each representing one animal. A nonparametric Mann–Whitney U test was used to compare quantitative data; *p, 0.05 and **p, 0.01. GraphPad Prism version 4 was used for statistical analysis.

## Results

### Prior encounter with adenovirus inhibits the induction of a transgene-specific primary immune response using the same vector

**Figure 1:**
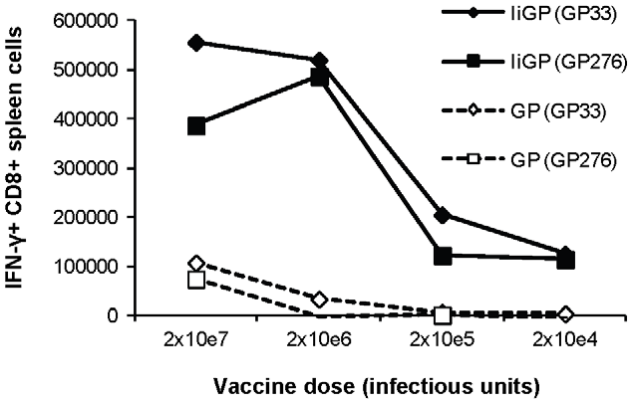
Dose-response correlations for Ad5-IiGP and Ad5-GP vectors. Groups of mice were vaccinated with the indicated vector doses and 14 days later the mice were sacrificed and the splenocytes were stimulated in vitro for 5 hrs. using the indicated epitopes. Antigen-specific CD8 T cells were enumerated using intracellular cytokine staining for IFN-γ.

Based on the fact that tethering of the transgenic antigen to invariant chain markedly improves the transgene-specific immune response, we hypothesized that the same degree of virus neutralization, which would completely prevent vaccination with a classical vector construct would still allow efficient priming with our optimized vector. As a first experiment to test this assumption we did a dose-response experiment, in which groups of mice were vaccinated with decreasing dose of Ad5- IiGP or Ad5-GP. Fourteen days later the transgene-specific CD8 T-cell response was measured. As can be seen in Fig. 1, a 100-fold reduction in inoculum, from the 2×10^7^ infectious units normally used for immunization to 2×10^5^ infectious units, still allowed the induction of a substantial transgene-specific CD8 T-cell response when we used Ad5-IiGP, whereas the same reduction in the dose of Ad5-GP completely eliminated an immune response. Thus, assuming as much as 99% neutralization of the incoming inoculum, the optimized vector should still induce a sizable CD8 T-cell response directed towards the inserted transgene.

**Figure 2:**
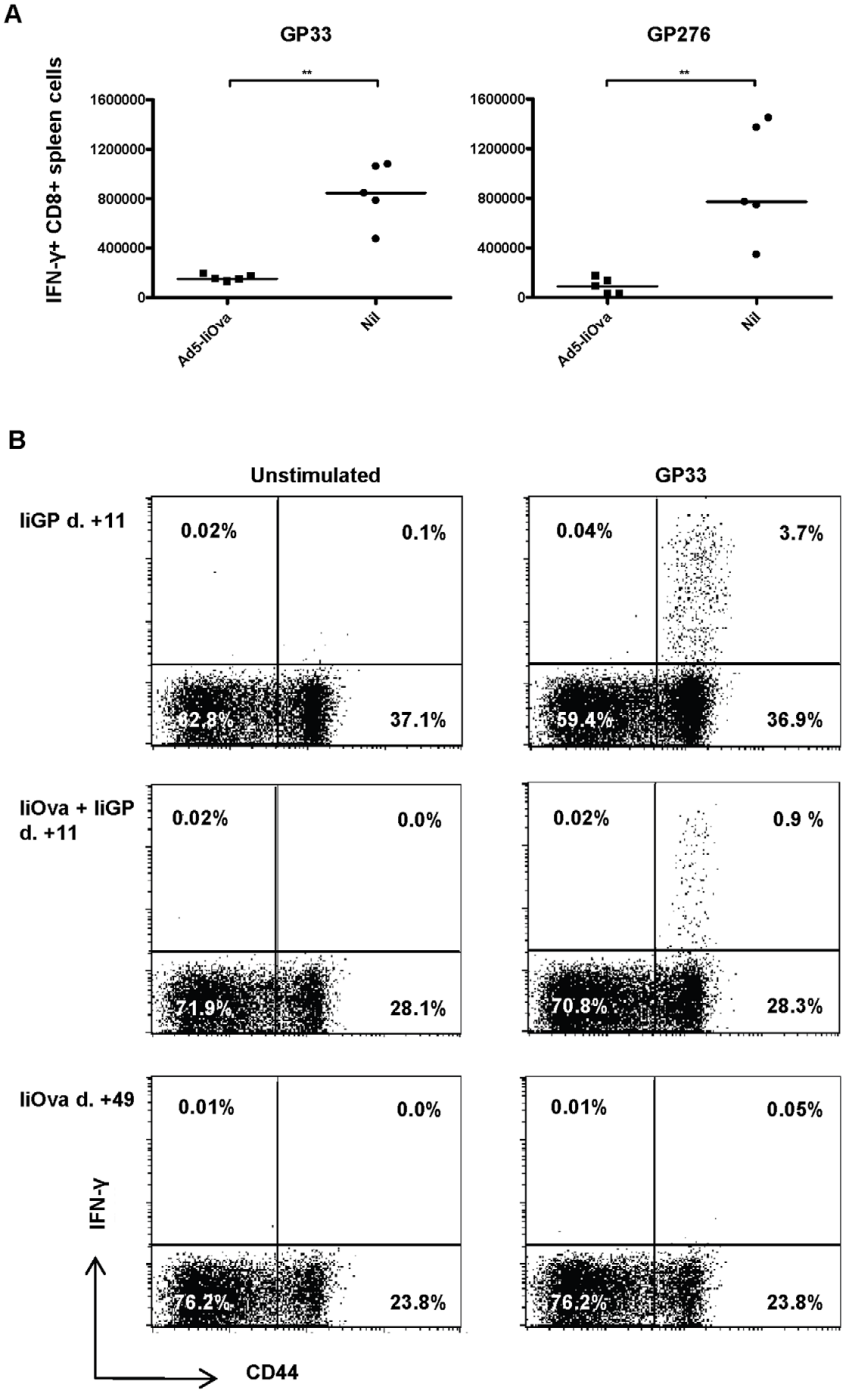
Prior encounter with adenovirus serotype 5 inhibits the induction of a primary transgene-specific CD8 T cell response using the same vector. Mice were initially vaccinated with either Ad5-IiOva followed by a second vaccination with Ad5-IiGP 40 days later. The GP-specific CD8+ T cell response was measured on days 11. (A) GP-specific CD8 T cell numbers in the spleen. B) Representative dot plots of gated CD8 T cells following in vitro stimulation in the presence or absence of GP33.

To directly test this prediction, mice were initially vaccinated with an irrelevant vector, Ad5-IiOva, followed by a second vaccination with Ad5-IiGP 40 days later. The GP-specific CD8 T-cell response was measured 11 days later and compared to the response in matched, vector-naïve mice. As expected (Fig. 2), the GP-specific CD8 T-cell response was clearly inhibited in mice previously exposed to Ad5. The inhibition of the vector induced immune response was more pronounced for the GP276 specific response resulting in a ∼9 fold reduction of CD8 T-cell numbers versus a ∼5 fold reduction in numbers of GP33 specific CD8 T cells (Fig. 2A;representative dot plots can be seen in Fig. 2B); for a more extensive analysis of the degree of inhibition, please see Fig. S1. Nevertheless, despite these reductions a distinct transgene-specific CD8 T-cell response was observed in the majority of vector immune mice (Fig. 2 and Fig. S1.)

### Inhibitory immunity primarily, but not exclusively reflects the presence of virus-specific antibodies

Having confirmed that previous encounter with Ad5 reduces the vaccine-induced response in most mice, we proceeded to examine the underlying mechanism(s). Both neutralizing antibodies and T cells have previously been implicated in the inhibition observed when vaccinating with adenoviral vectors in previously immunized mice [2], [3], and we therefore wanted to see if both mechanism were also relevant in our system. To address this issue, two complementary experimental set-ups were applied, which would separate the effect of antibodies from other specific immunological factors.

**Figure 3:**
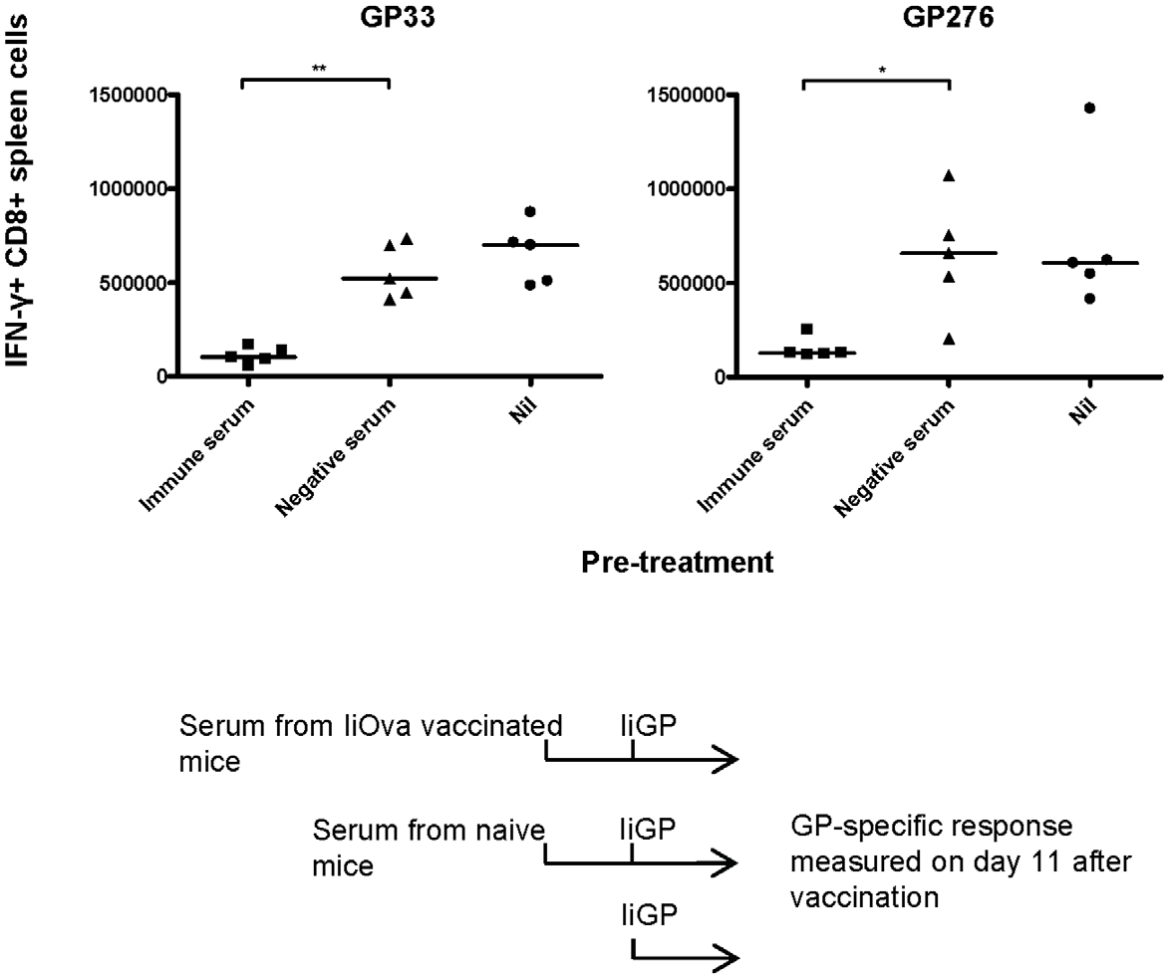
Transfer of serum from Ad5 immunized mice inhibits the induction of a transgene-specific CD8 T cell response by the same vector. Mice were vaccinated with Ad5-IiOva and 40 days later, serum (immune serum) was transferred to naïve mice so that each recipient received 1 ml serum. One day after serum transfer, recipient mice were vaccinated with Ad5-IiGP and the GP- specific CD8 T cell response was measured on day 11 after vaccination.

In the first set-up, mice were vaccinated with Ad5-IiOva, and 40 days later, serum was transferred to naïve mice so that each recipient received 1 ml serum in total (0.5 ml i.v. and 0.5 ml i.p.). As shown previously by Thomsen et al. [19], transfusion of this amount of serum should result in an antibody titer in the circulation of the recipients, which is about one third of the concentration in donor serum. One day after serum transfer, recipients were vaccinated with Ad5-IiGP and the GP specific CD8 T-cell response was measured on day 11 after vaccination. A group of mice receiving serum from naïve animals was included as a control. As can be seen in Fig. 3, the CD8 T-cell response was significantly inhibited in mice that received serum from previously immunized mice, while control serum had no inhibitory effect. The inhibition caused by serum transfer was not as pronounced as that seen in previously vaccinated mice, which may reflect of the lower antibody concentration mentioned above or that T cells also contribute to this phenomenon.

**Figure 4:**
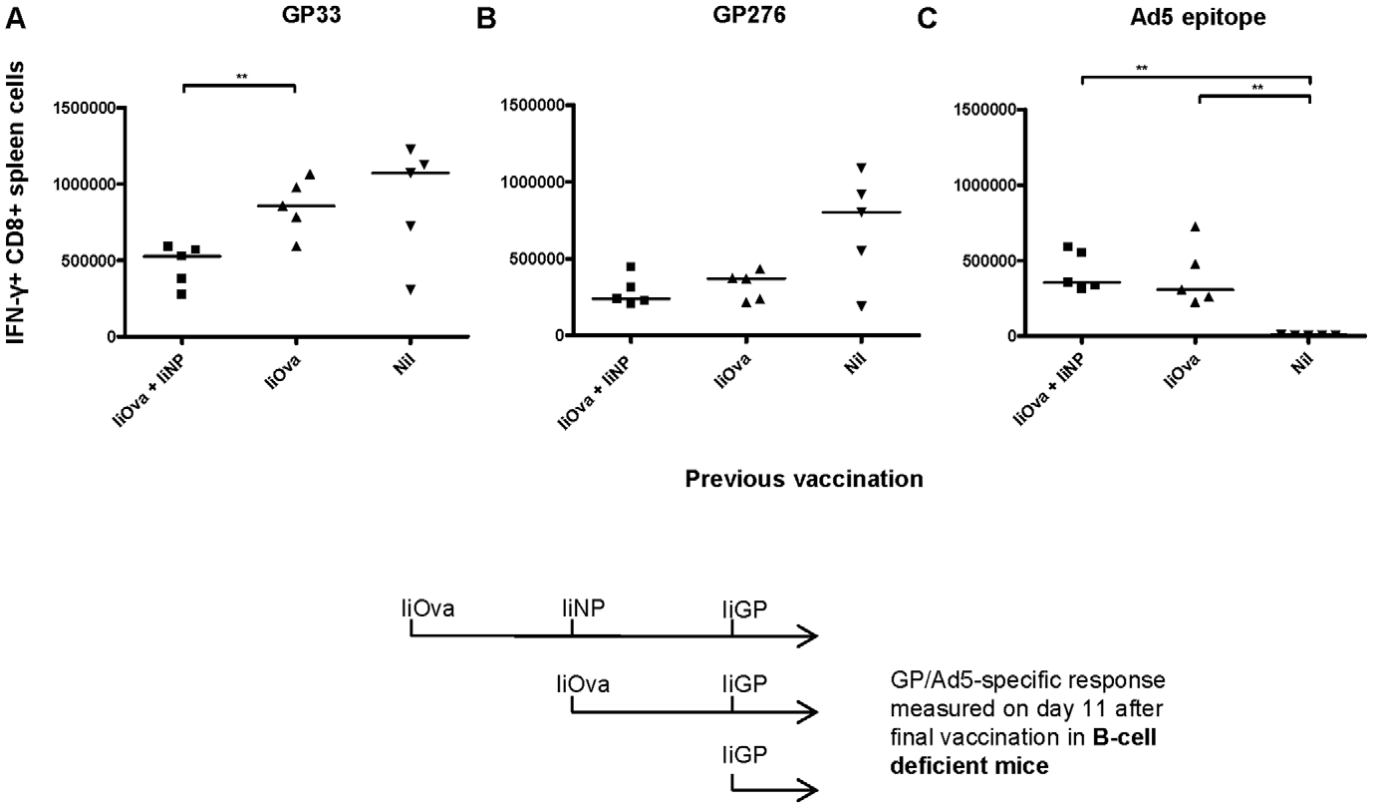
Prior encounter with adenovirus does not inhibit the induction of a transgene- specific CD8 T cell response by the same vector in B cell deficient mice. B cell deficient mice were vaccinated with Ad5-IiOva followed by a second vaccination with Ad5-IiGP or Ad5-IiNP on day 40. The group receiving Ad5-IiOva followed by Ad5-IiNP received a third vaccination with Ad5-IiGP 40 days after the second immunization. The GP- or Ad5-specific CD8 T cell response was measured on day 11 after the final vaccination (second or third). The experimental design and timeline is outlined above.

Complementary to the serum transfer approach, B cell deficient mice were vaccinated with Ad5-IiOva (or Ad5-IiGP) followed by a second vaccination with Ad5-IiGP or Ad5-IiNP on day 40. The group receiving Ad5-IiOva followed by Ad5-IiNP, received a third vaccination with Ad5-IiGP 40 days after the second immunization. The GP specific CD8 T-cell response was measured on day 11 after the final vaccination (second or third). The experimental design and timeline can be seen in Fig. 4. In the previous experiments involving inhibitory pre-immunity, we had also tested whether there would be a measurable T-cell response directed against an epitope in the adenoviral backbone. In those experiments we had observed very low, barely detectable responses in wt mice (data not shown); however, we wanted to see whether this response would be higher when humoral immunity was absent. Therefore, the Ad5-specific CD8 T-cell response was also measured on day 11 after the final vaccination.

In the absence of antibodies, Ad5 vaccination resulted in a normal, unimpaired GP33 specific CD8 T-cell response in vector immune mice (Fig. 4A), whereas the GP276 specific response tended to be somewhat inhibited in these mice, although the difference to naïve mice was not statistically significant (Fig. 4B). The latter finding could suggest that, at least in the absence of antibodies, there was also a weak T-cell based component involved in the inhibitory effect of prior vaccination with adenovirus. Consistent with this possibility, we also saw a significantly increased Ad5 specific CD8 T-cell response in pre-immunized mice compared to mice receiving only one vaccination with adenovirus (Fig. 4C).

Mice receiving 3 vaccinations with Ad5 vectors expressing three different exogenous antigens were included to see if the number of Ad5 specific CD8 T cells would increase proportionally to the number of vaccinations. At the time-point selected for analysis (day 11 after last vaccination), we did not observe a significant difference in the number of adenovirus-specific CD8 T cells between the groups receiving two or three vaccinations (Fig. 4C), but we did see a further inhibition of the GP specific CD8 T-cell response in the latter group (Fig. 4A+B), consistent with the notion that there might have been more Ad5 specific T cells present at the time of Ad5-IiGP vaccination in this group of mice. This adds further to the idea that Ad5 specific CD8 T cells might play a role in the inhibition, at least in mice unable to produce antibodies.

### Vaccination with adenovirus infected dendritic cells overcomes inhibitory immunity in previously vaccinated mice

**Figure 5:**
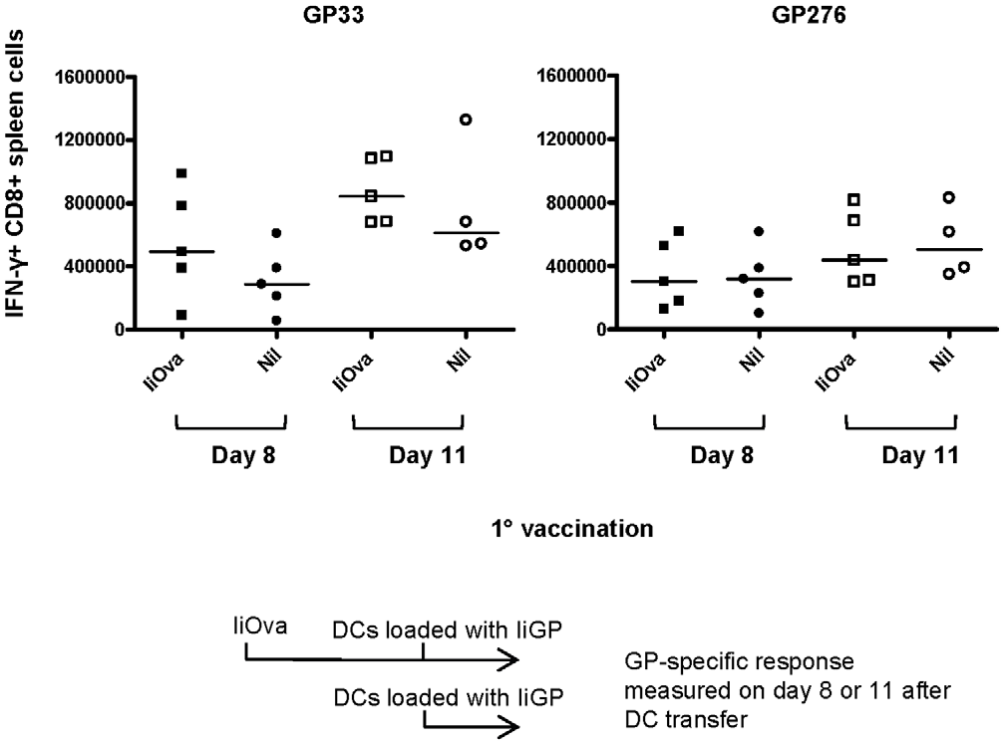
Vaccination with adenovirus infected dendritic cells overcomes inhibitory immunity in vector immune mice. Mice were immunized with Ad5-IiOva followed by a subcutaneous injection with 5×10^6^ Ad5-IiGP infected DCs 40 days later. The GP-specific CD8 T cell response was measured on days 8 and 11 after DC injection.

The above results point to antibodies as the major mechanism underlying the inhibition of vaccine-induced immune responses in vector-immune mice. If that is the case, masking the adenoviral vector inside dendritic cells (DCs) should circumvent the problem. To test this prediction, mice were immunized with Ad5-IiOva followed by a subcutaneous injection with Ad5-IiGP infected DCs 40 days later. The GP specific CD8 T-cell response was measured on days 8 and 11 after DC injection. Consistent with a major role for neutralizing antibodies, we saw no significant inhibition of the DC induced GP response for either of the epitopes tested in vector-immune mice compared to naïve controls (Fig. 5).

### The generation of vaccine-induced memory CD8 T cells is less affected than the expansion of primary effectors in Ad5-immune mice

**Figure 6:**
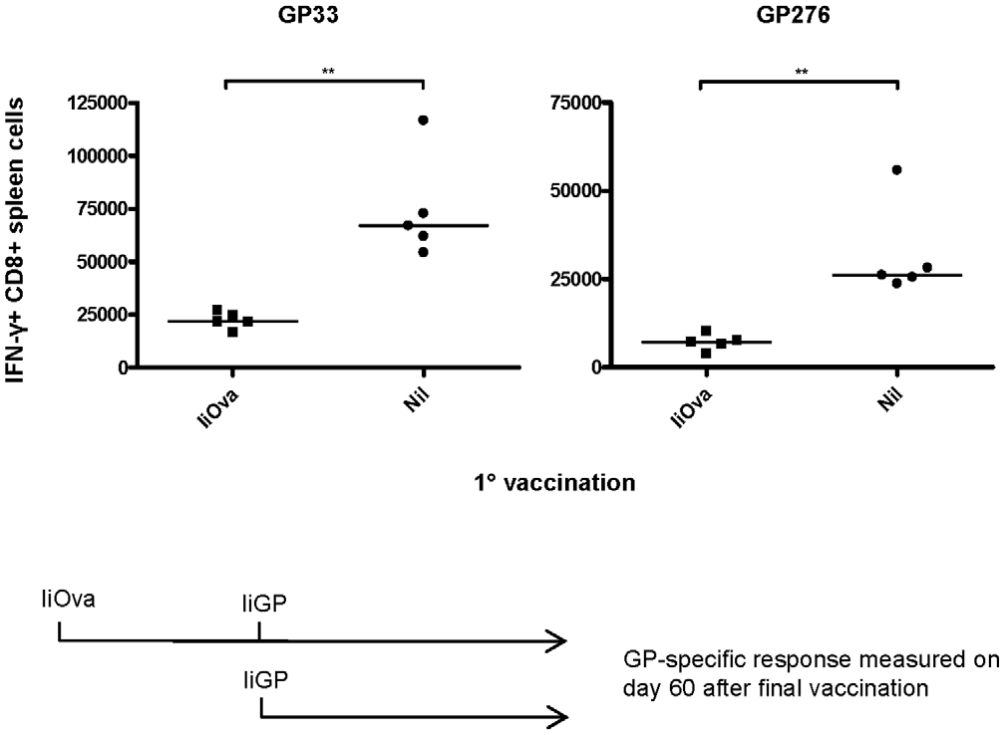
Vaccine induced generation of transgene-specific memory CD8 T cells is less affected than the expansion of primary effectors in Ad5-immune mice. Mice were initially vaccinated with Ad5-IiOva followed by a second vaccination with Ad5-IiGP 40 days later. The GP-specific CD8 T cell response was measured 60 days after the final vaccination.

For vaccination purposes, the induction of a strong memory response is the ultimate goal, and it was therefore of primary importance to investigate to which extent a reduction in the memory CD8 T-cell response would follow the reduction in the primary response. To study this, mice were initially vaccinated with Ad5-IiOva followed by a second vaccination with Ad5-IiGP 40 days later. The GP specific CD8 T-cell response was measured 60 days after the final vaccination. As previously seen with the primary effector response, numbers of memory CD8 T cells were significantly reduced in vector-immune animals (Fig. 6); however, it is interesting to note that the difference in numbers of memory CD8 T cells between previously vaccinated and naïve mice was only in the order of ∼3 fold compared to a difference ranging from 5–9 fold when it comes to the primary effector response (cf. Fig. 2). This indicates that even though the acute transgene-specific response is substantially inhibited in vector-immune mice, it seems that the memory response stabilizes at a level closer to what is induced in vector-naïve animals.

### Efficient recall responses despite reduced memory T-cell numbers in Ad5-immune mice vaccinated with the Ad5 vector

**Figure 7:**
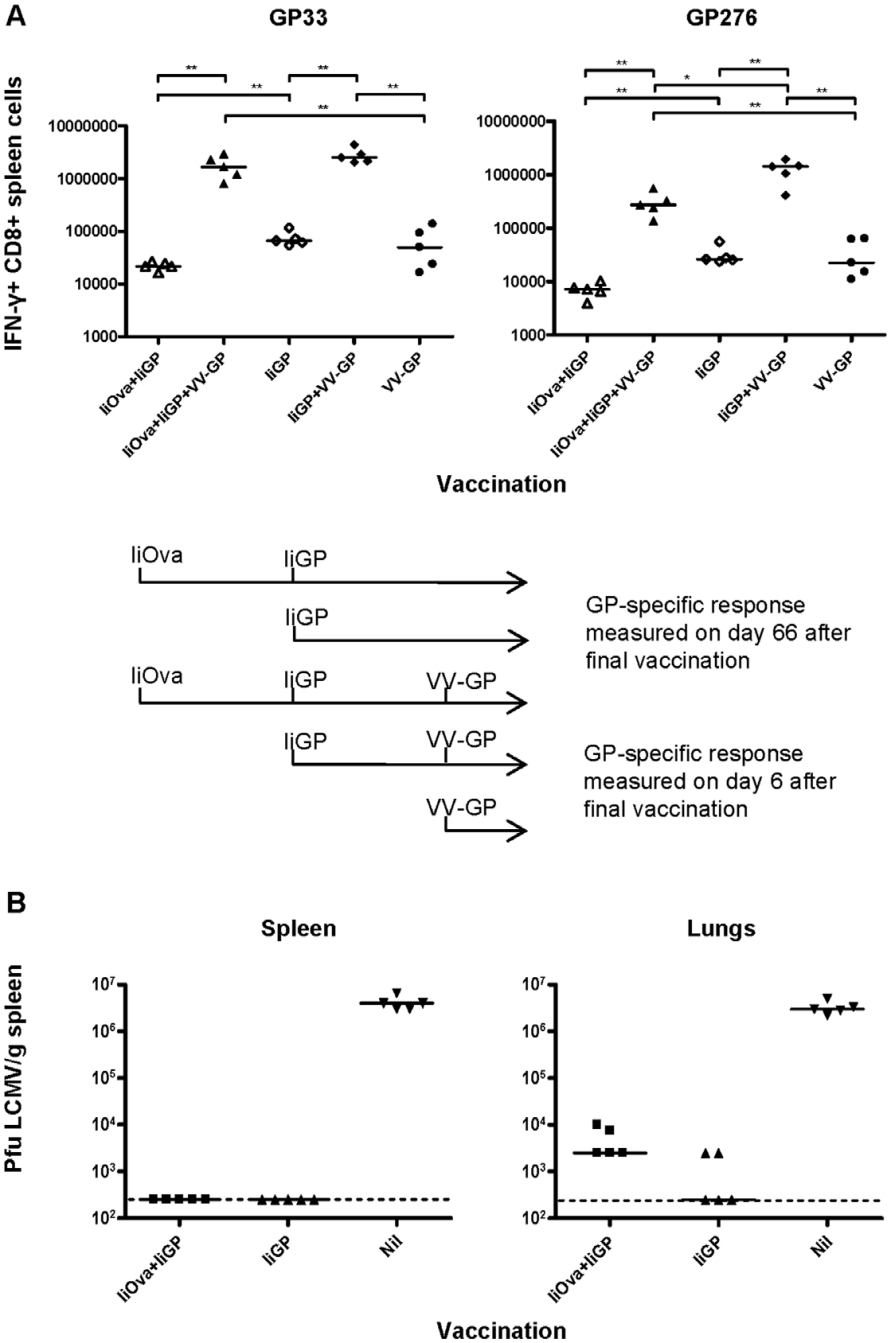
Efficient recall responses despite reduced memory T cell numbers in Ad5-immune mice vaccinated with the Ad5 vector. Mice were initially vaccinated with Ad5-IiOva, followed by a second vaccination with Ad5-IiGP on day 40. A) Sixty days after GP vaccination, the mice were challenged using vaccinia virus expressing GP of LCMV (VV-GP), and the GP-specific CD8+ T cell response was measured 6 days later. The experimental design and timeline is outlined above. B) Alternatively, mice vaccinated as described 60 days earlier were challenged with 10^6^ pfu of LCMVclone 13 i.v., and 5 days later spleen and lung virus titers were analyzed. The dotted line represents 250 pfu (limit of detection).

Having established that CD8 T-cell memory is somewhat impaired in mice with pre-existing vector immunity, it was pertinent to determine what this would mean in terms of reduced responsiveness to a viral challenge. For this reason, mice were initially vaccinated with Ad5-IiOva, followed by a second vaccination with Ad5-IiGP on day 40. Sixty days after the second vaccination, the mice were challenged using vaccinia virus expressing GP of LCMV (VV-GP), and the GP specific CD8+ T-cell response was measured 6 days later. The experiment also included groups receiving only Ad5-IiOva+Ad5-IiGP, Ad5-IiGP+VV-GP and Ad5-IiGP or VV-GP alone. The experimental design and timeline can be seen in Fig. 7.

Similar to the situation in mice primed when vector naive, the GP specific CD8 T-cell response of mice with impaired priming is also significantly boosted by vaccinia infection. Indeed, the GP33 specific recall response induced in these mice is nearly comparable to that observed in mice receiving only one vaccination with Ad5. The GP276 specific CD8 T-cell response can also be boosted in mice that have a repressed GP response before the challenge; however, in this case the number of secondary CD8 T cells generated is ∼5 fold lower than what is seen in mice with an uninhibited GP276 response before the challenge. Nevertheless, the most important finding in this experiment is that even though previously Ad5-immune mice generate fewer trangene specific memory CD8 T cells upon Ad5 vaccination, memory T cells are induced and will provide an accelerated response to a viral challenge.

To directly evaluate the functional capacity of the secondary effector CD8 T cells generated in mice with preexisting immunity towards the adenovector, we challenged mice primed with Ad5-IiGP in the presence or absence of vector immunity with a high dose (10^6^ pfu) of LCMV clone 13 i.v. and measured the viral load in the spleen and lungs 5 days later in these mice and unvaccinated controls. As can be seen in Fig. 7, infectious virus either could not be detected in vaccinated mice (spleen), irrespective of pre-existing vector immunity, or was found in minimal amounts only in mice with pre-existing immunity (lungs); this is in sharp contrast to unvaccinated mice, which harbor LCMV in high titers in both organ sites. Since we have previously demonstrated that cytotoxic CD8 T cells represent the key protective effector mechanism under these conditions [20], the latter results strongly support the concept that efficient and protective CD8 T-cell memory can be induced even in individuals with preexisting immunity to the adenoviral vector.

### Pre-existing immunity does not prevent secondary expansion of vaccine-specific CD8 T cells

**Figure 8:**
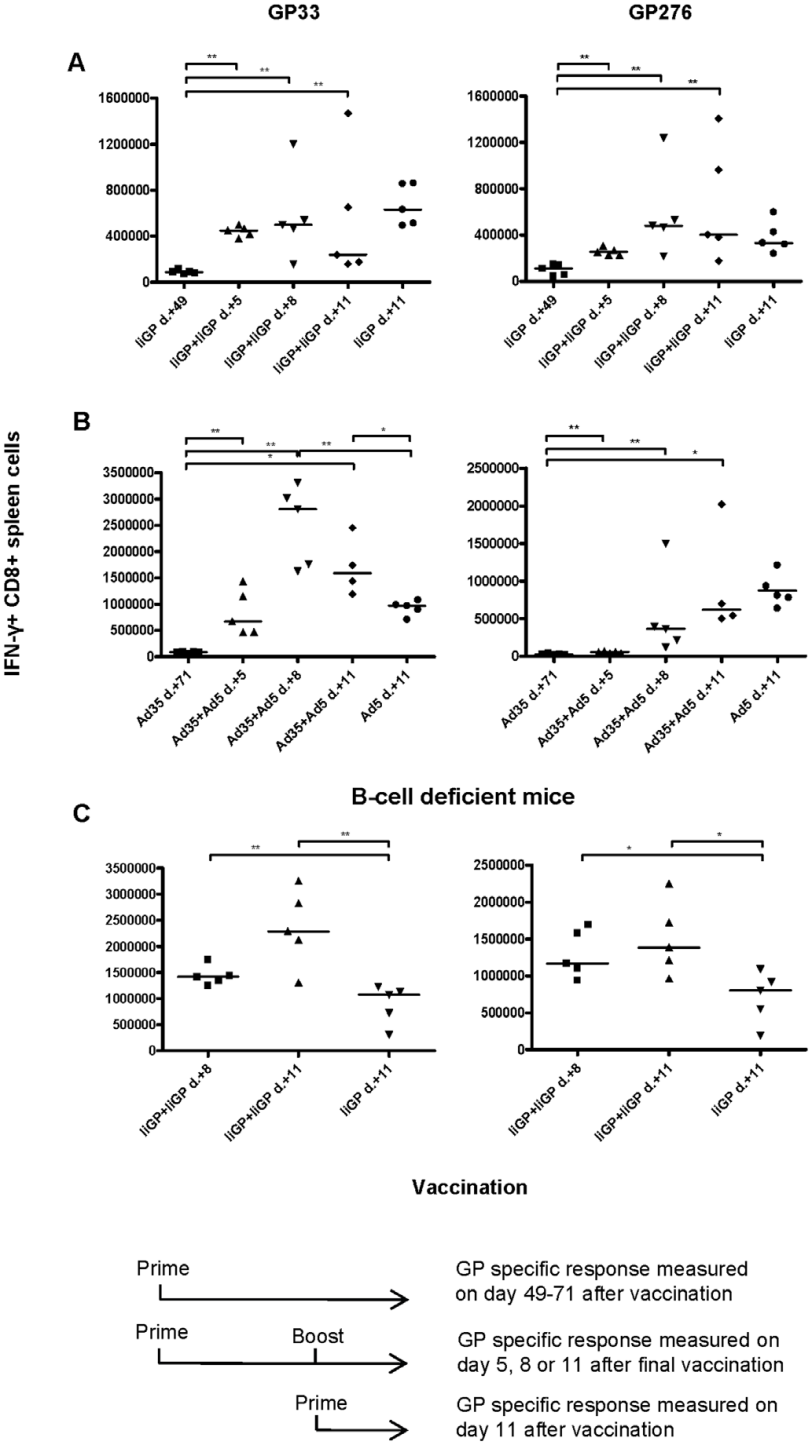
Pre-existing immunity reduces, but does not prevent secondary expansion of transgene-specific CD8 T cells. **A.** Mice were vaccinated with Ad5-IiGP, followed by a second vaccination with the same vector on day 40. The GP-specific CD8 T cell response was measured on days 5, 8 and 11 after the second vaccination; mice given a single vaccination with Ad5-IiGP 11 days earlier served as positive controls. **B.** Mice were primed using an Ad35-IiGP vector and about two month later given Ad5-IiGP. The GP-specific CD8 T cell response was measured on days 5, 8 and 11 after the second vaccination; mice given a single vaccination with Ad5-IiGP 11 days earlier served as positive controls. **C.** B-cell deficient mice were primed using Ad5-IiGP, followed by a second vaccination with the same vector about two months later. The GP-specific CD8 T cell response was measured on days 8 and 11 after the second vaccination; mice given a single vaccination with Ad5-IiGP 11 days earlier served as positive controls.

Having confirmed that the primary CD8 T-cell response is substantially inhibited in vector immune mice, whereas memory generation is not, we found it pertinent to investigate whether Ad5 induced secondary expansion would be inhibited. To address this question, mice were vaccinated with Ad5- IiGP, followed by a second vaccination with the homologous vector on day 40. The GP specific CD8 T-cell response was measured on days 5, 8 and 11 after the second vaccination. Contrary to expectations, a homologues vaccine booster induced a significant acute expansion of GP specific CD8 T-cell in pre-immunized mice, and the response showed the accelerated kinetics typical of a secondary response (Fig. 8A). Thus, significant T-cell expansion was observed on day 5 post vaccination; this is in contrast to primary responses induced by vaccination with this virus, in which case no response can be observed at this time point [21]. However, re-vaccination did not result in more effector cells being generated during recall than during primary vaccination. From this observation, it is would appear that there is some inhibition in pre-immune mice, but not enough to completely abolish a secondary Ad5 induced expansion.

In order to better appreciate the extent of the suppression of recall responses exerted in mice subjected to re-vaccination, two additional experiments were performed. In the first experiment (Fig. 8B), mice primed using a serologically unrelated Ad35 vector, Ad35-IiGP, were re-vaccinated using Ad5-IiGP vector about 2 month later, and the GP-specific CD8 T-cell response was measured 5, 8 and 11 days later. Despite the poorer T-cell priming known to follow the use of Ad35 vectors (ref), it is evident that an accelerated recall response against GP33 was observed following Ad5-IiGP vaccination, peaking at a level about twice that in unprimed mice - (regarding the response to GP276 it is our experience (personal observation) that Ad35 vaccination hardly causes any priming and the response kinetics is therefore not relevant in this context). Similarly, if B-cell deficient mice primed with Ad5-IiGP were boosted using the same vector (Fig. 8C), we also found a secondary response, which peaked at a level about twice that in unprimed mice. Thus. both these observation support the interpretation that a transgene-specific CD8 T-cell recall response can be induced in vector immune animals, but the induced response is reduced compared to that observed in mice lacking vector-specific antibodies.

### Memory cell expansion in previously vaccinated mice

**Figure 9:**
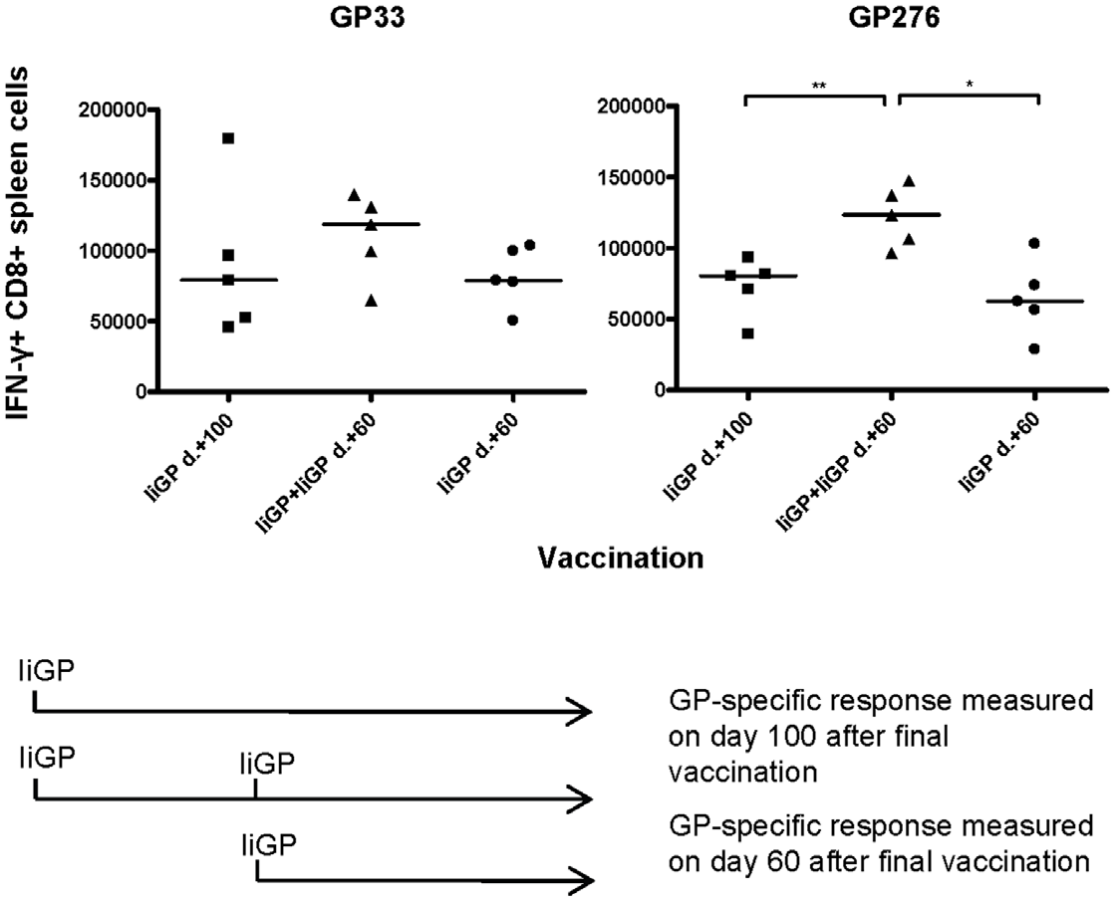
Memory cell expansion in previously vaccinated mice. Mice were vaccinated with Ad5-IiGP followed by a second vaccination with the same vector 40 days later, and the GP-specific CD8 T cell response was measured on day 60 after the final vaccination. Controls consisted of mice vaccinated once with the same vaccine either at the time of first immunization or simultaneously with the booster dose.

We also investigated memory cell expansion following a booster vaccination with Ad5-IiGP. Mice were vaccinated with Ad5-IiGP followed by a second vaccination with the same vector 40 days later, and the GP specific CD8 T-cell response was measured on day 60 after the final vaccination. Controls consisted of mice vaccinated once with the same vaccine either at the time of first immunization, or simultaneously with the booster dose. As can be seen in Fig. 9, numbers of GP276 specific memory CD8 T cells were significantly higher after two vaccinations compared to a single vaccination, indicating that it may be possible to expand the memory pool even in pre-immune mice. The induced GP33 specific secondary memory CD8 T-cell response showed the same tendency without being statistically significant (Fig. 9). This result is in line with observations regarding the primary CD8 T-cell response, both indicating that the acute expansion in pre-immune mice does not directly correlate with memory cell levels.

Adenovirus-primed CD8 T cells tend to be dominated by cells with an effector-memory like phenotype [22], [23]. Therefore, having found that GP specific memory CD8 T-cell numbers could be boosted in pre-immune wt mice using the same vector, it became of interest to see whether two Ad5 vaccinations would cause a further terminal differentiation of the induced memory cells. To this end, we measured the expression of the phenotypical markers CD27, CD127 and KLRG1 on the memory CD8 T cells generated after one or two vaccinations; combinations of these markers is commonly used to define different phenotypes of memory cells [24], [25]. However, the expression of all markers was similar irrespectively of whether the mice had been subjected to one or two vaccinations (Fig. S2), indicating that boosting of adenoprimed memory CD8 T cells is not associated with a significant change in the state of their differentiation.

### On the role of vaccination route

**Figure 10:**
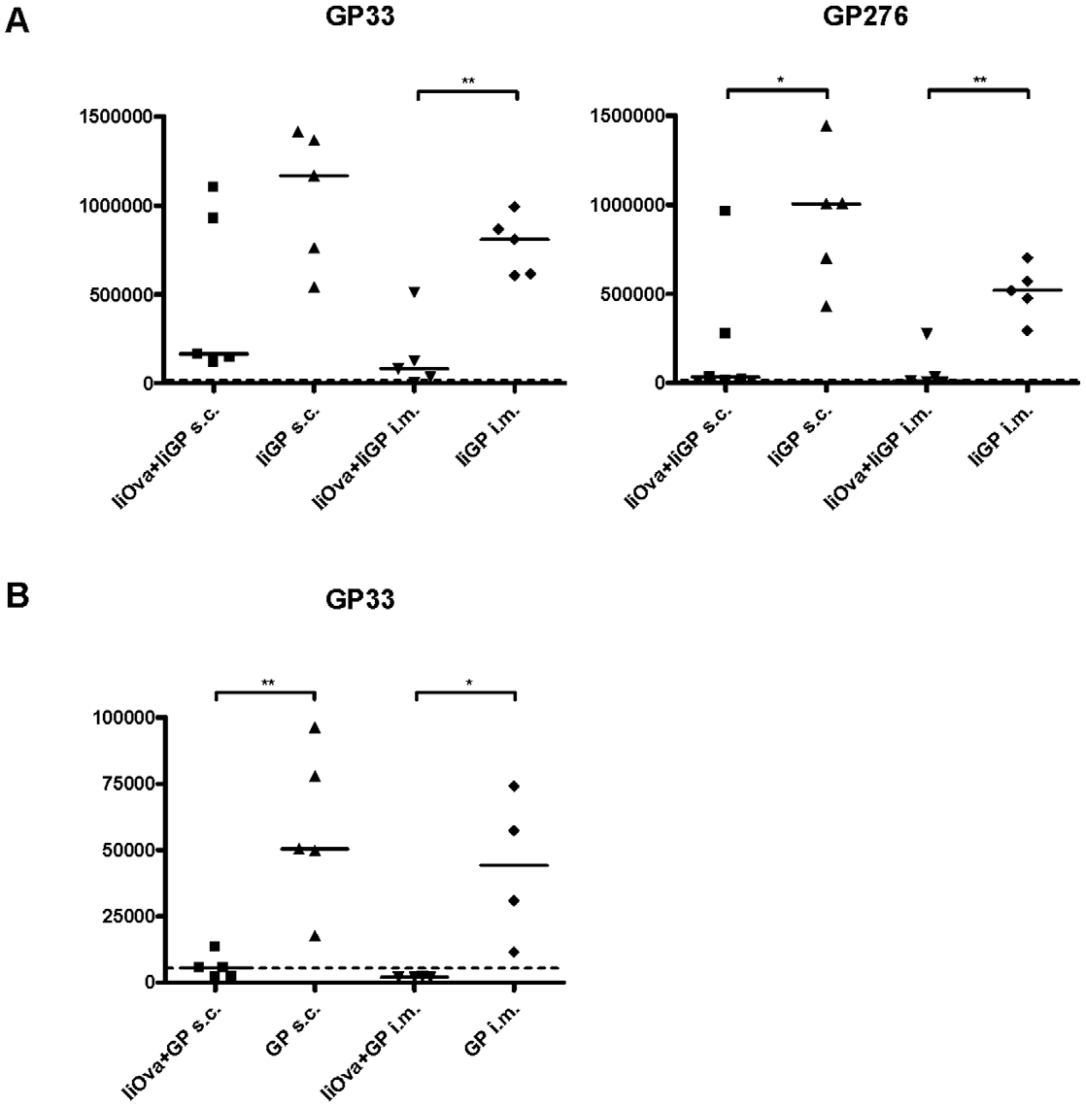
Influence of vector configuration and route of vaccination. Groups of mice were either vaccinated with Ad5-IiOva or left untreated. Two months later, mice from both groups were vaccinated i.m. or s.c. with Ad5-IiGP or Ad5-GP, and the transgene-specific CD8 T-cell response was evaluated. Results for GP276 in mice vaccinated with Ad5-GP have not been not been included as this vector construct does not cause a clearly detectable CD8 T-cell response even in vector-naïve mice. Dotted lines denote limit of detection for IFN-γ^+^ CD8 T cells as determined by analysis of unstimulated cells.

Since it is a major finding of our study that we tend to observe much better CD8 T-cell priming in vector-immune animals than it is reported in the literature, we sought to explain this discrepancy. One obvious factor could be the way in which we present the antigen. Thus tethering of the transgene to invariant chain obviously would seem to play a major role, cf. Fig. 1. However, also the fact that we used s.c. immunization as opposed to i.m. vaccination used in most published studies, could be relevant. To address the relative importance of these factors, we performed an experiment in which two large groups of mice were either vaccinated with Ad5-IiOva or left untreated. Two months later, mice from both groups were vaccinated i.m. or s.c. with Ad5-IiGP or Ad5-GP, and the transgene-specific CD8 T-cell response was evaluated (Fig. 10). Similar results were obtained using both epitopes except in the case of mice vaccinated with Ad5-GP, in which mice a GP276-specific cannot be detected no matter the route of vaccination. Irrespective of route of vaccination and vector construct, the induced CD8 T cells seemed to be subject to the same relative reduction in vector -immune mice as compared to vector-naïve animals. However, because the tethered construct was much more immunogenic than the conventional construct, a sizable T-cell response could still be observed in vector-immune mice given the former, whereas the transgene-specific T-cell response in vector–immune mice given the conventional construct borders on the level of detection. Regarding the role of route of vaccination, no major difference was observed; however, due to the fact that i.m. vaccination was associated with a lesser immune response than s.c. immunization even in vector-naïve mice, responses tended to be lower also in i.m. vaccinated vector-immune mice.

## Discussion

This study focuses on how prior encounter with adenovirus impacts a transgene specific CD8 T-cell response induced by vaccination with the same adenoviral vector. Earlier studies have shown that Ad5 induces a humoral and cellular immune response that may inhibit the induction of an immune response using the same vector [2], [3], [12]. Following these discoveries, there have been several studies focusing on changing the serotype of the virus used for vaccination or modifying the hexon proteins on the surface of the Ad5 capsid [13], [26]–[30]. However, having devised a way to improve the stimulatory capacity of transgenes expressed from adenoviral vectors, we found it pertinent to revisit the subject of inhibitory immunity in pre-immune hosts and to clarify the full extent of inhibition on the generation of memory and secondary effectors cells, since this would have important implications for the design of future vaccines.

Using our optimized vector system, we still observed an inhibitory effect on the transgene specific CD8 T-cell response when the mice have pre-existing immunity to the Ad5 vector, and the same phenomenon was observed for the Ad35 vector (data not shown). As to the underlying mechanism, although published data indicate that antibodies are important as mediators of the inhibition observed in mice with pre-existing immunity to the viral vector [2], [3], T cells have also been implicated and, notably, complementary analysis in B-cell deficient mice has not previously been performed. Strongly supporting a role for antibodies, we observed not only that transfer of serum from Ad5 vaccinated donors caused a marked reduction in the number of antigen specific CD8 T cells induced by Ad5 vaccination in the recipients, but also that the transgene specific response was not significantly reduced by prior encounter with Ad5 in previously vaccinated B cell deficient mice, and that re-vaccination with an Ad5 vector expressing the same antigen induced robust secondary responses in the latter mice. Finally, the inhibitory effect of pre-existing immunity could be overcome by vaccinating mice with in vitro Ad5 loaded DCs, that mask the adenovirus particles from recognition by antibodies.

However, pre-existing antibodies do not explain everything. Thus, in B-cell deficient mice we found that after two and three Ad5 vaccinations, numbers of Ad5 specific CD8 T cells were substantially increased, a phenomenon not observed in wt mice (data not shown). Moreover, mice with an increased number of adenovirus specific CD8 T cells after three Ad5 vaccinations had a significantly lower number of GP33 specific CD8 T cells compared to mice vaccinated only once. The GP276 specific response showed the same tendency, but in this case there was no statistical significance. Since antibodies can be ruled out as a cause of inhibition in these mice, the results support the idea that T cells might also contribute to the inhibitory effect of prior encounter, at least in a context where antibodies are not able to neutralize the virus. Pertinent to this hypothesis, it should be noted that Sumida et al. [3] have claimed an inhibitory effect of an Ad5 induced CD8 T-cell response based on adoptive transfer of primed splenocytes to naïve recipients. We have not been able to reproduce this observation (data not shown). However, this could be a matter of variations in virus dose (10^10^ vs. 2×10^7^) and/or mouse strain (BALB/c vs. C57BL/6) employed. Another study that points to some degree of T-cell involvement in the inhibition associated with pre-existing immunity was performed by Osada et al. [9] who observed that an E1, E2b and E3 deleted vector induced an immune response of the same magnitude in naïve and previously vaccinated mice. These authors hypothesized that this was due to a significantly lower production of late proteins such as the hexon protein after infection of DCs in vivo, which might mean that DCs are not cleared so rapidly by CD8 T cells allowing the immune response to develop in spite of the presence of adenovirus-specific CD8 T cells. If this hypothesis holds true, there seems to be some involvement of T cells in mediating the inhibitory effect of pre-existing immunity to adenoviral vectors.

Having confirmed that pre-existing immunity to the vector markedly interferes with the induction of a primary effector CD8 T-cell response, we found it pertinent to investigate whether the clonal expansion of memory T cells was repressed to the same degree, since the available number of memory cells would be of primary importance in regard to the ability to mount efficient recall responses upon antigen challenge. We observed that the number of memory CD8 T cells was significantly lower in animals with pre-existing Ad5 immunity; importantly, however, the memory cell level in these mice was not reduced to the same degree as the primary effector response. This observation led us to investigate the ability of the remaining memory CD8 T cells to respond to challenge with live virus expressing the antigen used for vaccination. We found that the memory cells induced in the face of inhibitory immunity responded well to the viral challenge, and only the GP276 specific response was significantly lower in mice with a repressed memory level. Furthermore, irrespective of the state of vector immunity at the time of vaccination, vaccinated mice were able to rapidly control a high dose challenge with highly infectious LCMV clone 13. These findings have obvious and important implications regarding the use of the Ad5 vector in immune individuals, because they indicate that even though the primary Ad5 induced response may be repressed in vector-immune individuals, these may still be able to mount efficient recall responses upon viral re-challenge.

Secondary immune responses have a lower threshold for activation, a faster kinetics, and result in the generation of higher numbers of antigen specific cells compared to primary immune responses. As expected from this, we saw a significantly smaller impact of pre-existing immunity on the ability of the Ad5 vector to induce a secondary immune response. Notably, the magnitude of the recall response was not higher than a normal primary response, but the response did exhibit the accelerated kinetics of a secondary immune response.

Having observed a diminished inhibitory effect of pre-existing immunity on memory cell formation in Ad5-immune mice, we asked whether CD8 T-cell memory could be boosted by a second vaccination with the same virus vector. We observed that the number of GP276 specific memory CD8 cells was higher in mice vaccinated twice with the Ad5-IiGP vector, which indicates that even though the magnitude of the effector response is not increased after two vaccinations, some expansion of the memory CD8 T-cell pool may still occur. Numbers of GP33 specific memory CD8 T cells also tended to be higher in mice vaccinated twice with Ad5; however, in this case the difference was not statistically significant. Notably, the phenotype of antigen specific CD8 T cells induced by two vaccinations was similar to the phenotype of memory cells induced by only one vaccination, which is important for vaccine purposes where it is important to sustain an expanded memory population without driving the involved cells towards replicative senescence.

In the current study, we found much better CD8 T-cell priming in vector- immune animals than has previously been reported in the literature [1], [4], [13]. This predominantly reflects the improved immunogenicity of our construct resulting from tethering of the transgene product to invariant chain. Using the optimized construct, dose-response analysis revealed that even assuming 99% neutralization of applied inoculum, a transgene-specific CD8 T-cell response would be induced, which matches that in vector-naïve mice given an optimal dose of a classical vector construct. Also the route of vaccination seems to play a role. Thus, despite a similar relative reduction in the CD8 T-cell response following s.c. and i.m. vaccination of vector-immune mice, the fact that s.c. vaccination overall tends to induce a stronger response, leaves room for a slightly better response in vector-immune mice when vaccinated s.c. compared to i.v.

We find it to be of particular importance that quite substantial recall responses could be observed in recipients with pre-existing immunity towards the vector at the time of first vaccination. This could indicate that our optimized Ad5 vector can be used even in Ad5- immune individuals, albeit with slightly reduced efficiency. If this holds generally true, and we have results suggesting that it is the case also for Ad35, this would have important implications for the design of vaccine strategies utilizing adenoviral vectors, since it removes some of the need for a substantial combinatorial library of different vectors in order to sustain the immune response, e.g. in the case of cancer patients where prolonged immune pressure is likely to be important for successful therapy [31], [32]. Instead, it would be possible to combine a limited number of potent vectors, which each could be used more than once and still sustain an efficient effector CD8 T-cell response for a significant period of time.

## Supporting Information

Figure S1 **Inhibition of the primary immune response is present up to 10 months after initial vaccination.** Mice were initially vaccinated with Ad5-IiOva followed by a second vaccination with Ad5-IiGP 1, 2, 4, 6, 8 or 10 months later. The GP specific CD8 T cell response was measured 11 days after the second vaccination. For each pre-immunized animal, the measured response was compared to the average of responses in a matched control group of 4–5 previously naïve mice, and from this comparison, percent responsiveness was calculated. The spread in the degree of inhibition matches with a similar spread in antibodies titers of previously adenoexposed mice. Thus, in a group of 14 randomly chosen mice, we observed neutralizing antibody titers varying between hardly detectable and 1∶640 (median = 1∶40).
(TIF)

Figure S2 **Expression of the phenotypical markers CD27, CD127 and KLRG1 on transgene specific memory CD8 T cells generated after one or two vaccinations with Ad5.** Mice were vaccinated as described in fig. 9 and the expression of CD27, CD127 and KLRG1 on the GP specific memory CD8 T cells was measured on the indicated days. Representative contour plots on gated GP33-specific (IFN-γ^+^) CD8 T cells are depicted to the right.
(TIF)
