# Supplementary figures and images for: Pre-Existing Vector Immunity Does Not Prevent Replication Deficient Adenovirus from Inducing Efficient CD8 T-Cell Memory and Recall Responses
Source: PLoS One. 2012 Apr 13;7(4):e34884. doi: 10.1371/journal.pone.0034884 (PMC3326056; doi:10.1371/journal.pone.0034884)

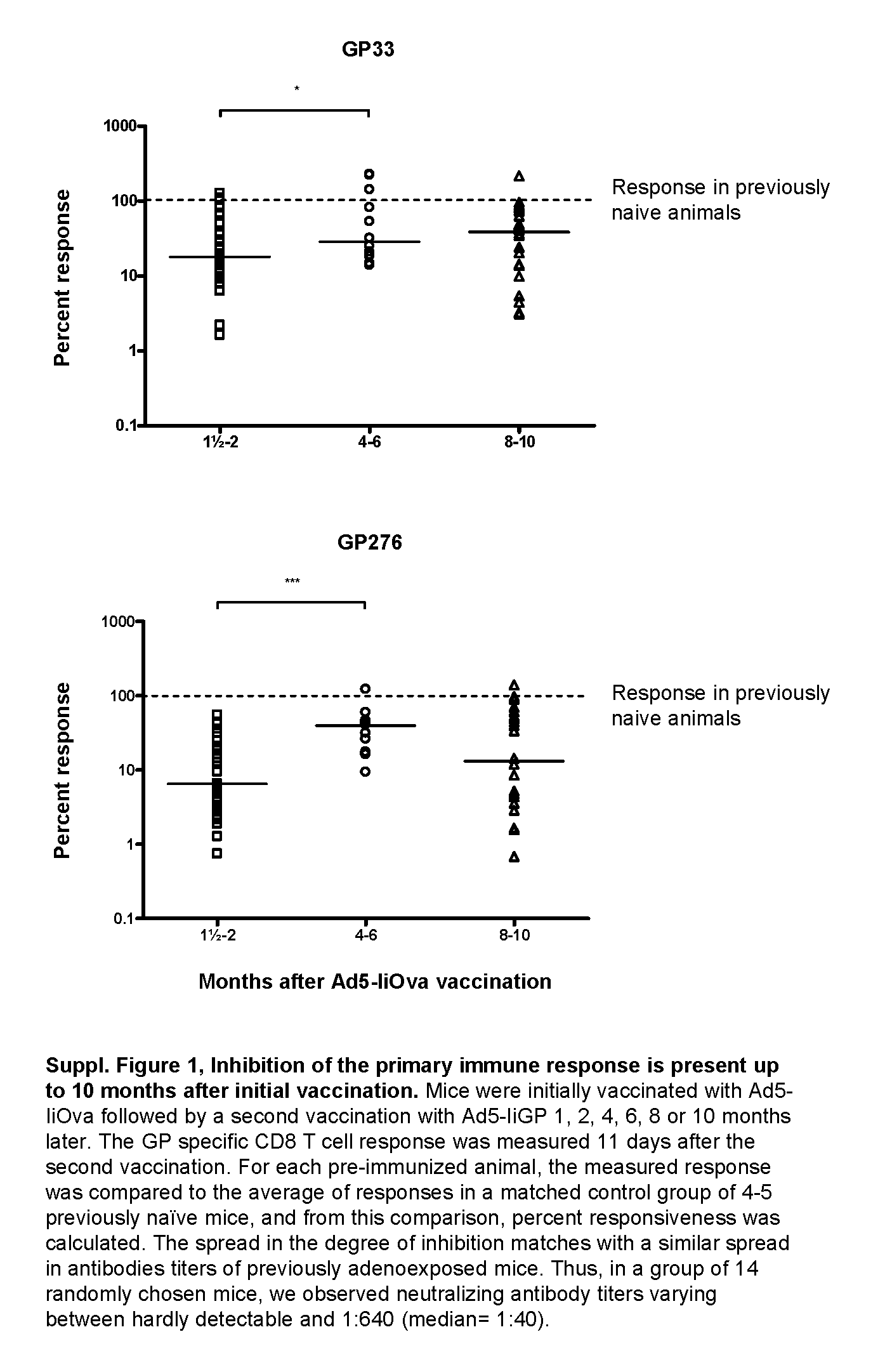

Supplement: Figure S1 — Inhibition of the primary immune response is present up to 10 months after initial vaccination. Mice were initially vaccinated with Ad5-IiOva followed by a second vaccination with Ad5-IiGP 1, 2, 4, 6, 8 or 10 months later. The GP specific CD8 T cell response was measured 11 days after the second vaccination. For each pre-immunized animal, the measured response was compared to the average of responses in a matched control group of 4–5 previously naïve mice, and from this comparison, percent responsiveness was calculated. The spread in the degree of inhibition matches with a similar spread in antibodies titers of previously adenoexposed mice. Thus, in a group of 14 randomly chosen mice, we observed neutralizing antibody titers varying between hardly detectable and 1∶640 (median = 1∶40). (TIF) [file pone.0034884.s001.tif]

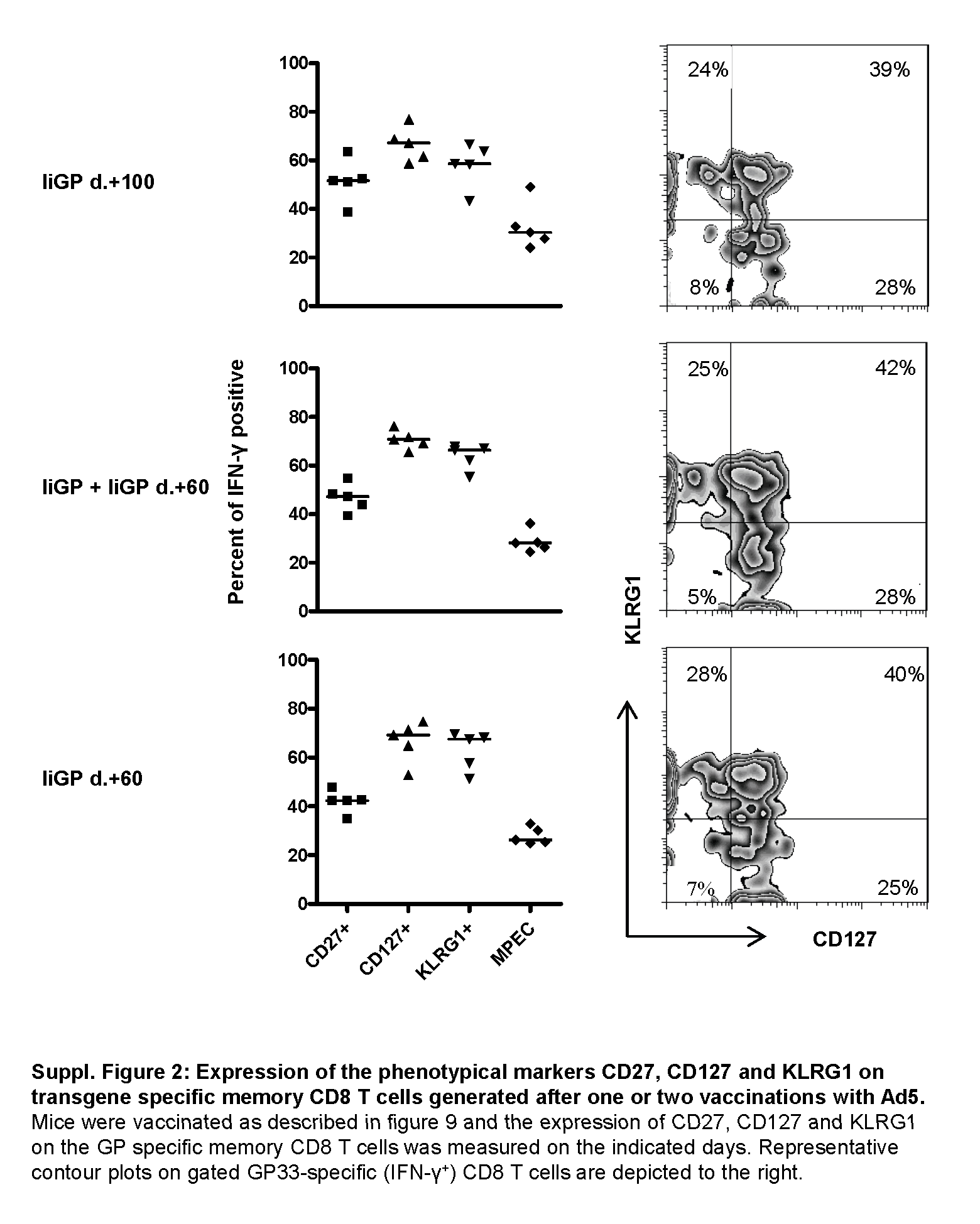

Supplement: Figure S2 — Expression of the phenotypical markers CD27, CD127 and KLRG1 on transgene specific memory CD8 T cells generated after one or two vaccinations with Ad5. Mice were vaccinated as described in fig. 9 and the expression of CD27, CD127 and KLRG1 on the GP specific memory CD8 T cells was measured on the indicated days. Representative contour plots on gated GP33-specific (IFN-γ+) CD8 T cells are depicted to the right. (TIF) [file pone.0034884.s002.tif]
